# Evaluation of specific germ cell genes expression in mouse embryonic stem cell-derived germ cell like cells treated with bone morphogenetic protein 4 in vitro

**Published:** 2018-08

**Authors:** Maryam Gholamitabar Tabari, Seyed Gholam Ali Jorsaraei, Mohammad Ghasemzadeh-Hasankolaei, Ali Asghar Ahmadi, Neda Mahdinezhad Gorji

**Affiliations:** *Infertility and Reproductive Health Research Center, Health Research Institute, Babol University of Medical Science, Babol, Iran*.

**Keywords:** Bone morphogenetic protein, Embryonic stem cells, Germ cells, Embryoid body

## Abstract

**Background:**

Bone morphogenetic protein 4 (BMP4) is a significant signaling molecule that involves in initiating of differentiation and performs multifunctional effects on embryonic stem cells (ESCs) and embryos.

**Objective:**

The goal of the present study was to evaluate an in vitro differentiation model of mouse embryonic stem cells into germ cells, using BMP4.

**Materials and Methods:**

in this experimental study, we used Oct4-GFP mouse ESCs to form embryoid body (EB) aggregations for two days. Then, single cells from EB were cultured for four days with BMP4. Using MTT assay and gene expression levels for evaluation of Mvh and Riken by real-time RT-PCR of six concentrations, 12.5 ng/ ml BMP4 was determined as an optimized dose. Then, the expression level of *Fkbp6*, *Mov10l1*, *4930432K21Rik*, *Tex13*, *Mvh*, *Scp3*, *Stra8*, *Oct4* were evaluated. Flow cytometry and immunostaning were used to confirm the findings of the real-time RT-PCR.

**Results:**

In the +BMP4 group, the genes encoding Riken (p≤0.001) and *Mvh* (p≤0.001) were found to be increased with significant differences compared with the control group. *Mov10l1* (p=0.22), *Tex13* (p=0.10), *Fkbp6* (p=0.90), *Scp3* (p=0.61) and *Stra8* (p=0.08) were up-regulated without significance differences compared with control group. Flow cytometry analysis showed that the mean number of *Mvh*-positive cells in the +BMP4 group was greater when compared with ESCs, -BMP4 and EB groups (p=0.03, p≤0.001, p=0.02, respectively).

**Conclusion:**

Down-regulation of *Oct4*, expression of germ cells genes and meiosis markers expression raise this hypothesis that ESCs were differentiated by BMP4, and may be introduced into the first meiosis as germ cell-like cells.

## Introduction

Germ cells (GCs) are generated from primordial germ cells (PGCs) during embryogenesis and form spermatogonia and oogonia during gametogenesis (1). Embryonic stem cells (ESCs) that are derived in vitro from the inner cell mass (ICM) of the blastocyst are considered as a valuable source for gamete production. These are pluripotent stem cells with self-renewal and unlimited proliferative ability (2) and can accumulate and form cell clusters or embryoid bodies (EBs).

Under certain conditions, ESCs can differentiate into all three embryonic germ layers, and germ cells. Gonocytes, either in females enter meiosis by inhibiting mitosis, or, in males enter the G1 stage by inhibiting meiosis in prespermatogonia (3). A short-term transmitting factor in male gonads is produced, which terminates cell cycle at G1, and prevents the cells from entering meiosis (4). Whether a meiosis-inducing factor (5) or an autonomous event is required for GCs to enter meiosis (6) is still a controversial issue. Significant results from the genital ridge and epiblast-derived PGCs in vitro indicated that these cells respond to signals such as the leukemia inhibitory factor (LIF), stem cell factor (SCF) (7), tumor necrosis factor (TNF) (8) and retinoic acid (RA) (9) for proliferation. One of the molecular signals that play an important role in the onset of differentiation, and has multiple effects on embryos and stem cells are bone- morphogenetic protein 4 (BMP4). BMP4 is a family member of transforming growth factor -β (10). When ESC- derived EB was exposed to BMP4 for 3-10 days, expression of developed PGC markers was significantly increased (11). The various effects of BMP4-mediated differentiation are dependent on the culture method (12), its concentration (13) and the exposure time (14). Reproductive biology studies on mammalian embryonic development face two major problems: limited access to cellular material, and lack of specific markers that are expressed only in germline or in GCs during in vitro differentiation. Furthermore, genetic and morphological similarities between ESCs and PGCs make it difficult to distinguish between them in cultures during in vitro differentiation. In mice, over 53 genes are involved in the regulation of the cell cycle (15). 

*Fkbp6*, *Mov10l1*, *4930432K21Rik*, and *Tex13* are specific markers that are expressed in GCs but are either not expressed or show very low expression in ESCs (16). *Mov10l1* is an X-linked and GC-specific gene in mouse spermatogonial cells (17). *Tex13* is an X-linked gene expressed during GC specification at the onset of spermatogonial differentiation (18). *Fkbp6* gene was identified from RNA in mouse testicular cells by real-time RT-PCR. (19). Mutations in this gene are associated with male infertility. *4930432K21Rik* is a novel gene with unknown function that is expressed in PGCs, testis, spermatozoa, and oogonia (20). Few studies have examined expression levels of these genes in differentiation using in vitro inducers. 

In order to evaluate the expression of these genes (*Fkbp6*, *Mov10l1*, *4930432K21Rik*, and *Tex13*) in in vitro, here we designed an in vitro model for GCs differentiation from mESCs, using BMP4.

## Materials and methods

**Animals**

In this experimental study, 5 NMRI mice (8-10 wk old) weighting: 35-40 gr were housed in a 12 hr light/ dark cycle (6:00 AM to 6:00 PM). Mice were given food and watered ad libitum.

**Study design**

There are three groups in this experimental study. 1-ESCs differentiated using EB method (2 days) then EB aggregation singled and cultured with 12.5ng/ml BMP4 for 4 days as (+BMP4) 2- ESCs differentiatiated using EB method (2 days) then EB aggregation singled and cultured without BMP4 for 4 days as control group (-BMP4) 3- EB aggregation in day 2 of culture as (EB).

**Culture medium**

**MEF medium**

This is a knock out-DMEM (Dulbecco’s modified Eagle’s medium) (Gibco, Paisley, UK) containing 15% FBS (Biowest, USA), Pen/Str/Glu 100 units/ml (Gibco), 100 mMnon-essential amino acids (Gibco), 0.1 mM 2-Mercaptoethanol (Gibco, Paisley, UK). 

**ESC culture medium**

Knock out-DMEM (Dulbecco’s modified Eagle’s medium) (Gibco, Paisley, UK) containing 15% Knock out-SR (Gibco), Pen/Str/Glu 100 units/ml (Gibco, Paisley, UK), 100 mMnon-essential amino acids (Gibco, Paisley, UK), 0.1 mM 2-Mercaptoethanol (Gibco, Paisley, UK), 1000 IU/ml LIF (Chemicon). 

**EB medium**

This is ESC medium without LIF**. **

**ESC Differentiation medium**

ESC culture medium that supplemented with 12.5 ng/ml BMP4 (R & D, USA) and 15 ng/ml bFGF (Invitrogen, USA).

**The culture of mouse embryonic fibroblasts (MEFs)**

ESCs need co-culture with feeder layer for proliferation. Cultures from E13.5 mice fetus were used to generate mouse embryonic fibroblasts (MEFs) as feeder layer in this study. The procedure briefly explained: put some female mice with some male mice together in the cage (two females with a male mouse) to mate. The morning after mating vaginal plug was checked and pregnant mice were identified and were scored as E0.5. After 13-day pregnant mice were sacrificed to extract embryos. The embryos were isolated and washed, and then head, heart, and liver were separated from the embryo. The removed organs were crushed using an 18-gauge needle and then cultured in MEF media. After two passages, MEF cells were ready to be inactive by 10 g/ml mitomycin C (Sigma, Germany( 1.5-2 hr for ESCs culture as a feeder layer. We used passages from 2 to 4 in work.

**Culture and passage of mouse ESCs (mESCs)**

The OG2 (△PE-GFP) ESC line (Max Planck, Germany) was used in this study. It was cultured as described briefly below. The mESC line was maintained on mitomycin C-treated MEFs in 0.1% gelatin-coated 25 cm^2^ flasks in ESC medium. Undifferentiated ESCs were cultured at 37^o^C, 5% CO^2^, and 95% humidity and the medium was renewed daily. Seventy-two hr after primary culture, when the colony size increased, cultures were trypsinized and expanded at a ratio of 1:3 on fresh feeder cells. The medium was changed every day. 

**EB formation**

ESCs were dissociated with 0.25% trypsin-EDTA (Invitrogen) and collected in EB medium. In order to separate MEFs from ESCs, we used the ability of MEFs that re-attach faster than ESCs to a tissue culture plate to omitted MEF. After two rounds of reseeding (about 30-40 min), the supernatant containing the ESCs was extracted. These cells used for EB formation. It was induced in two ways: 1) Hanging drop culture prepared with a cell suspension containing 150–200 ESCs per 25 l of mouse EB differentiation media; and 2: suspension culture where ESCs were seeded at a density of 1.5×106 cells per cm2 in an EB differentiation media in a non-attachment 10 cm^2^ bacterial dish for EB formation. 

We evaluated both methods in our laboratory and did not find any differences in EB formation between two methods. Since a large amount of cells was required, the suspension culture method was used to produce enough cells. After 2 days, EBs were dissociated and digested by collagenase IV treatment (0.01%) in order to obtain cell suspensions, before being filtered and were seeded in a gelatinized dish at about 20.000 cells per cm^2^. To determine the optimized dose, BMP4-induced cell differentiation for 4 days (BMP4 exposure) was examined in six concentrations (1, 5, 12.5, 25, 50, 100 ng/mL). Expressions of two candidate genes (*Mvh*, Riken) were evaluated by real-time RT-PCR. 

In addition, the MTT assay was performed to evaluate the replication and cell proliferation at different concentrations of BMP4. Cells were fed daily with ESC differentiation medium supplemented with BMP4 and bFGF 15 ng/ml for four days. 

**RNA isolation and real-time RT-PCR**

Total RNA was isolated from ESCs, BMP4 treated Putative GCs (+BMP4), non-BMP4 treated Putative GCs (-BMP4), two days EB (EB2), and somatic tissues of the testis and brain using the RNA Isolation Kit (Roche, Germany). Genomic DNA contamination was eliminated using DNase I. RNA quality was determined using a Nanodrop 2000c (Thermo Scientific). cDNA was prepared in a total volume of 10 μL using a cDNA synthesis kit (Takara, Japan) according to the manufacturer’s protocol. 

Target gene expressions were normalized based on the mouse housekeeping gene Hprt. Gene transcripts were assessed using SYBR Green I PCR Master Mix (Applied Biosystems) containing 150 nmol each of the forward (F) and reverse (R) primers (Table I). Real-time RT-PCR analysis was performed using the ABI 7300. Relative quantification of gene expression was calculated using the 2-△△Ct method. Three technical replicates were used for each real-time RT-PCR reaction; a no-template blank served as a negative control. In all reactions, mouse testis and brain were used as positive and negative controls, respectively. 

**Immunofluorescence staining**

*Mvh*-positive cells appear and can develop into PGC-like cells. This test was conducted in accordance with the protocol of the antibody manufacturer briefly, the cells cultured in two-wells chamber slide, were fixed in 100% methanol (chilled at -20^o^C) at room temperature for 5 min. Then, the cells were heated in antigen retrieval buffer (100 mM Tris, 5% [w/v] urea, pH 9.5) to 95^o^C for optimal performance of certain antibodies. Coverslips were heated at 95oC for 10 min. The cells were incubated for 10 min in PBS containing 0.1-0.25% TritonX-100 (ICN) for cell permeabilization. 

Subsequently, the cells were incubated with 1% BSA, 22.52 mg/mL glycine in PBST (PBS+ 0.1% Tween 20) for 30 min to block non-specific binding of the antibodies. The Cells were then incubated in diluted *Mvh* primary antibody (1:100, Anti-DDX4 / *Mvh* antibody Abcam 13840, UK) in 1% BSA in PBST in a humidified chamber overnight at 4oC. Then, the cells were incubated with the secondary antibody (1:100-1:400 goat anti-rabbit IgG-PE: sc-3739, USA) in 1% BSA for 1 hr at room temperature in the dark, followed by incubation with 0.1-1 μg/mL DAPI (Sigma, USA) for 1 min. Coverslips were mounted with a drop of mounting medium. Finally, the cells evaluated under an inverted fluorescence microscope (Canada smart, Canad).

Testis tissue samples were used for this test. Abcam protocol describes briefly; Slides were allowed to reach room temperature. Slides were washed 3 times in TPBS (PBS-tween), each time for 5 min before being immersed in Triton X-100 (0.2% for a cytoplasmic antigen) for 20 min. Blocking was performed in 10% normal serum with 1% BSA in TPBS for 2 h at room temperature, followed by incubation with *Mvh* primary antibody (1:100) diluted in TPBS with 1% BSA overnight at 4oC in the dark. Fluorochrome-labeled secondary antibody (goat anti-rabbit IgG-PE) diluted in TBS with 1% BSA was applied to the slide and the slide was incubated for 1 hr at room temperature in the dark. The coverslip was mounted using a compatible mounting medium. 

**Flowcytometry**

Four groups of cells were analyzed with flowcytometry. +BMP4, -BMP4, EB2, and undifferentiated ESCs. Methods (Abcam protocol) are described briefly; the cells were fixed before intracellular staining to ensure the stability of soluble antigens or antigens with a short half-life. Then, they were fixed in 0.01% formaldehyde for 10-15 min. 100 μL detergent-based permeabilizing agent Triton ×100 (0.1-1% in PBS) was added and incubated in the dark at room temperature for 15 min. 0.1-10 μg/ml of the *Mvh* (Abcam 13840) primary antibody was added andincubated for at least 30 min at 4^o^C in the dark. The fluorochrome-labeled secondary antibody (goat anti-rabbit IgG-PE: sc-3739) was diluted in 3% BSA/PBS at the optimal dilution (1:100-1:400) and incubated for at least 20-30 min at 4^o^C in the dark. They were resuspended in ice cold PBS, 3% BSA, 1% sodium azide. Secondary antibody IgG-PE was detected by FL1 channel of FACS Calibur TM flowcytometer (BD Biosciences, USA) and the percentage of positive cells was measured by FlowJo 7.6 software

**Ethical consideration**

The maintenance and care of experimental animals complies with National Institutes of Health guidelines for the humane use of laboratory animals (MUBABOL.REC.1393.7). 

**Statistical analysis**

All experiments were independently repeated at least three times. Data are presented as mean±SD. Statistical analysis was determined using ANOVA, Tukey. All statistical tests were performed using SPSS (Statistical Package for the Social Sciences, version 22.0, SPSS Inc, Chicago, Illinois, USA) software. p<0.05 was regarded as significant. 

**Table I:** Primer sequences for real-time RT-PCR

**Gene**	**Forward Primer**	**Reverse Primer**
*Oct4 *	TGTTCCCGTCACTGCTCTGG	TTGCCTTGGCTCACAGCATC
*Scp3 *	GCAGTCTAGAATTGTTCAGAGCCAGA	TCCAAACTCTTTATGAACTGCTCGTG
*Mvh*	GGAGAGAGAGCAAGCTCTTGGAGA	TGGCAGCCACTGAAGTAGCAA
*Stra8 *	GACGTGGCAAGTTTCCTGGAC	TTCTGAGTTGCAGGTGGCAAA
*Tex13 *	GCCACAGGAAGACCGAATGAG	TCTCTGCCTTTTCAGGGGATA
*Fkbp6 *	CCCCTCATCCCGCCAAATG	TGCCAAACTCCCTCTCAGTTG
*Mov10l1 *	CGCTGTGACGAGTACAGTG	CTGACAACCCTTTGCTAGAGTTT
*4930432K21Rik *	AGAGAGTCGGAAGACAGCTCA	CAGGGGGACCAGCTCTTTG
*Hprt *	GTTAAGCAGTACAGCCCCAAA	AGGGCATATCCAACAACAAACTT

## Results

**Figure 1:**
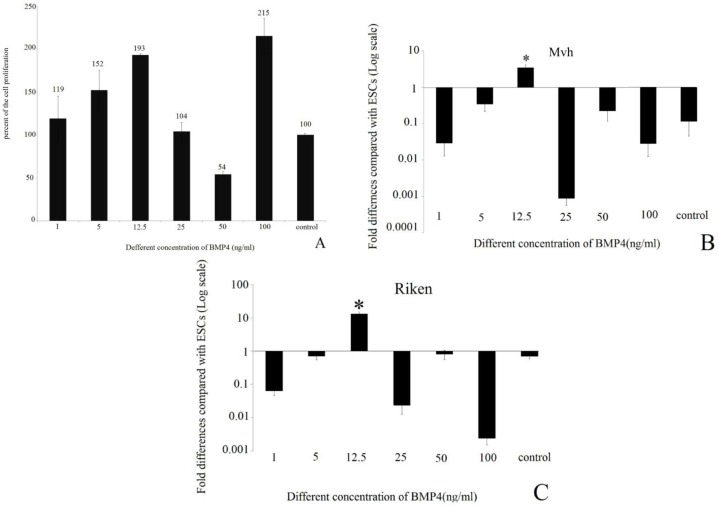
Diagrams of MTT assay and real-time RT-PCR from different concentrations of BMP4 to determine effective doses (A). MTT assay to evaluate the replication and cell proliferation in different concentrations including 1, 5, 12.5, 25, 50,100 ng (B, C) comparison of expression level of *Riken* and *Mvh* known as germ cell marker with real-time RT-PCR in different concentrations including 1, 5, 12.5, 25, 50,100 ng/ml.* p<0/05

The results of the MTT assay for replication and cell proliferation and real-time RT-PCR of *Mvh* and Riken genes to determine the optimal dose of BMP4, showed that the dose of 12.5 ng/ml is more appropriate than other doses (Figure 1A-C).

**Morphological evaluation**

**Figure 2:**
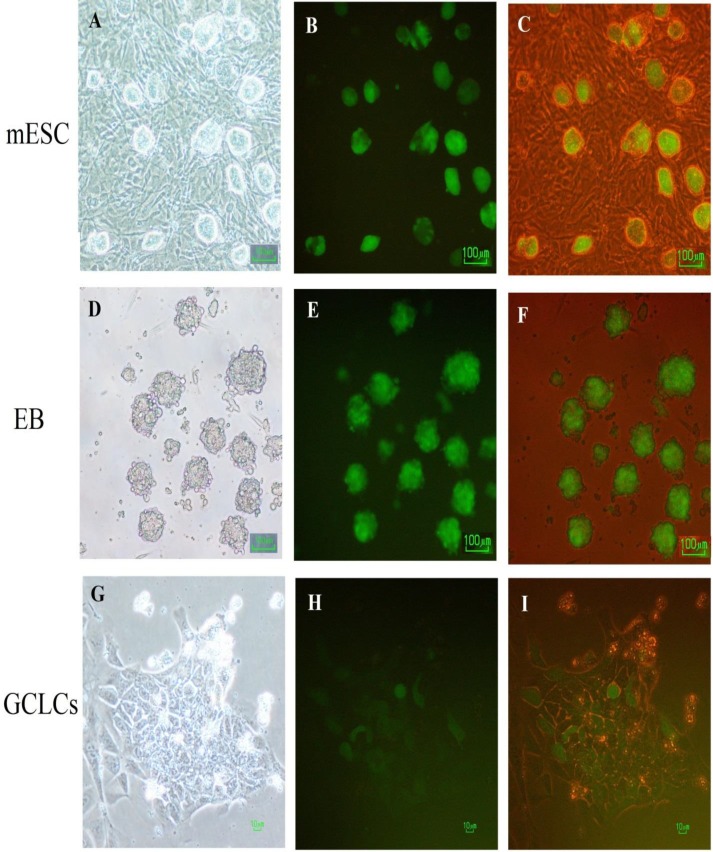
Morphological assessment of Oct4-GFP embryonic stem cell (ESC) colonies, day 2 of EB culture (EB 2) and ESCs-derived germ cell-like cells (GCLCs). (A) Bright field image of Oct4-GFP ESCs colonies growing on an embryonic fibroblast feeder layer, (B) Fluorescence image of Oct4-GFP ESC colonies with *Oct4* expression indicated in green (C) Merged fluorescent and bright field images of Oct4-GFP ESCs. (D) Bright field image of EB colonies growing after 2 days in suspension culture in the bacterial plate. (E) Fluorescence image of EB colonies with *Oct4* expression indicated in green. (F) Merged fluorescent and bright field images of EB colonies. (G) Bright field image of a Putative GC after 4 days culture with BMP4 without feeder cells in ESC differentiation medium (H) Fluorescent image of (G) image with *Oct4* expression indicated in green. (I) Merged fluorescent and bright field of (G) image

ESC colonies (Figure 2 A-C) and EB aggregation in day two (EB2) (figure 2 D-F) has been shown. MTT assay and expressions of two candidate genes (*Mvh*, Riken) showed that among with six dose of BMP4, 12.5 ng/ml was more effective than others compared with control group. Morphological evaluation of these cells showed that EB cells that were attached to the bottom of the dish formed colony-like collections and changed to round-shaped cells (Figure 2, G-I).

**Expression of GC-specific genes in in vitro-derived germ cell-like cells (GCLCs)**

Pluripotency and known GC marker expression (*Fkbp6*, *Mov10l1*, *4930432K21Rik*, and *Tex13*) as well as *Oct4*, *Mvh*, *Scp3*, *Stra8*, and Hprt (mouse housekeeping gene) were determined by quantitative real-time RT-PCR in five groups +BMP4, -BMP4, EB2, brain and testis. Comparisons were made with ESCs. Quantitative RT-PCR results indicated that gene expression differed between the groups. In the +BMP4 group, the genes encoding *4930432K21Rik* (p≤0.001) and *Mvh* (p≤0.001) were found to be increased with significance differences compared with those in the -BMP4 group.

**Figure 3:**
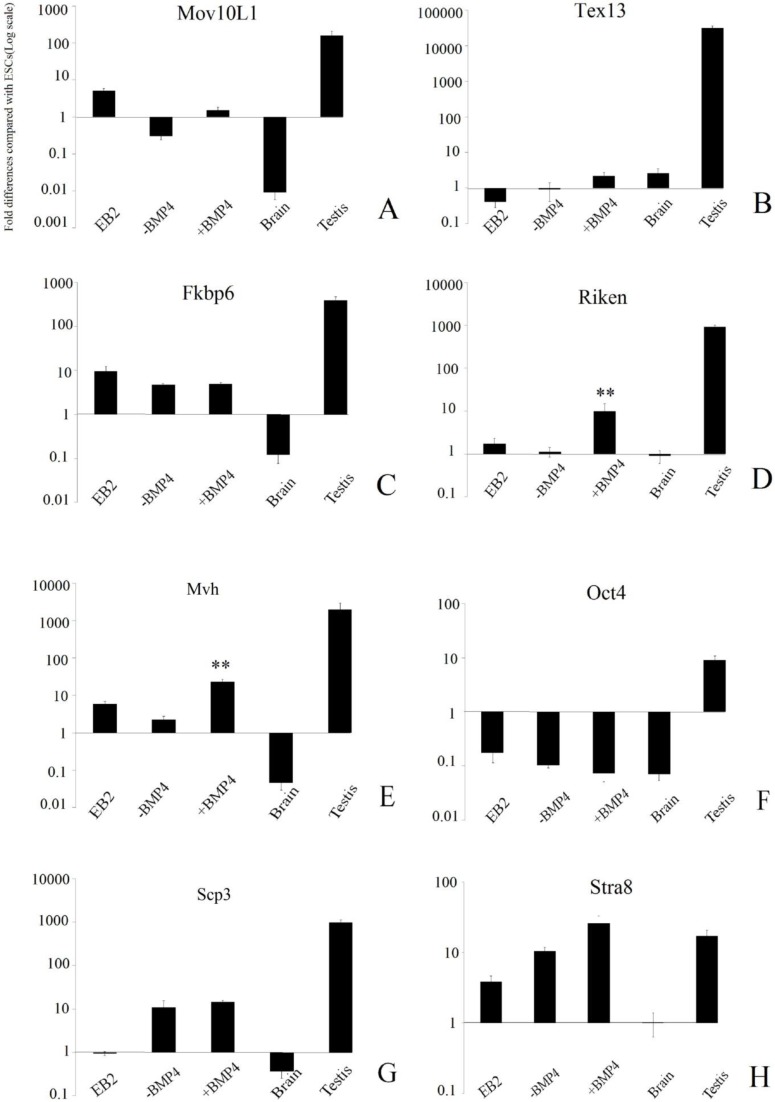
The expression pattern of a number of germ cell (GC)-specific genes in study groups. (A) expression level of *Mov10l1 *(B) *Tex13* (C) *Fkbp6* (D) *Riken* (E) *Mvh* (F) *Oct4* (G) *Scp3* (H) *Stra8 *in EB2,-BMP, +BMP4, brain, testis. The amount of the undifferentiated mESC is indicated as 1

**Figure 4:**
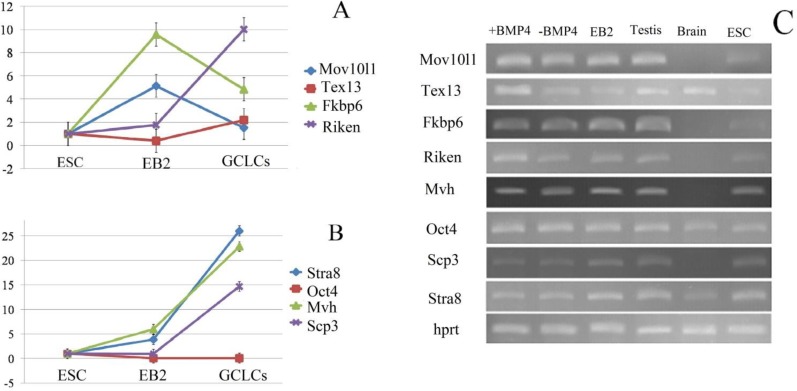
Comparison of the expression level of GC-specific genes in each study groups (A). The expression level of four germ cell-specific genes *(Fkbp6, Mov10l1, 4930432K21Rik, Tex13)* in ESCs, EB2, and germ cell-like cells (GCLC) after BMP4 treatment. (B) four general germ cell gene and pluripotency factor (*mvh* , *scp3*, *stra8*, and *Oct4*) in ESCs, EB2, and germ cell-like cells (GCLC) after BMP4 treatment. (C) Expression of germ-cell-specific genes. RNA was prepared from GFP- positive ESCs, +BMP4, -BMP4, EB2, brain and adult testis, and processed for real-time RT-PCR. *Hprt* expression was evaluated as a housekeeping gene for control

Also, *Mov10l1* (p=0.22), *Tex13* (p=0.10), *Fkbp6* (p=0.90), *Scp3* (p=0.61) and *Stra8* (p=0.08) were up-regulated but did not reach significance when compared with those in the -BMP4 group (p>0.05). *Oct4* expression was down-regulated when compared with its levels in ESCs among all study groups (p<0.05; Figure 3, 4). All GC-specific genes were found to be expressed at low to moderate levels in putative GCs. *Fkbp6*, *Mov10l1*, *4930432K21Rik*, and *Tex13* exhibited very low or no expression in brain tissues. In addition, these genes were expressed at moderate-to-high levels in adult testes. 

We found that the mRNA expression of Riken was higher as compared with that of other genes in in vitro derived putative GCs. Its expression was approximately 6.6-fold higher as compared with that of *Mov10L1*, 2 fold higher as compared with that of *Fkbp6*, and 4.7-fold higher as compared with that of *Tex13*. 

**Figure 5:**
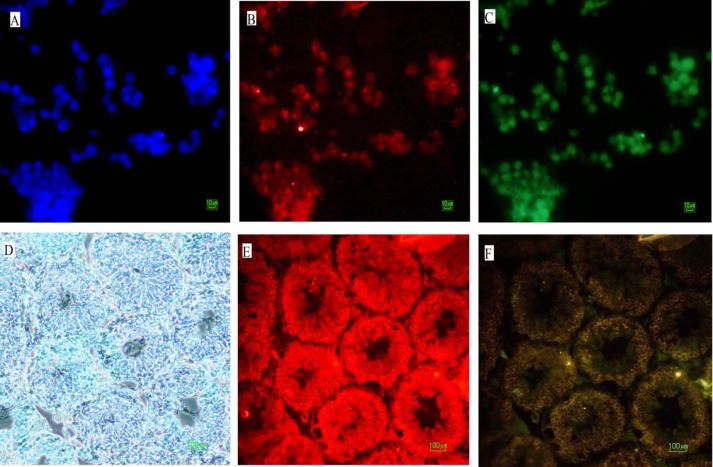
Immunofluorescence staining in study groups. (A) nuclei were stained with DAPI (in blue), (B) anti- *Mvh* antibody – as a GC Markers (in red) and (C) Oct4-GFP expression (in green). (D) the section of immunofluorescence staining of mouse adult testis in bright field, (E) anti *Mvh* antibodyas a GC Marker (in red) and (F) in dark field) The light of the microscope is off and the fluorescent light is on(.

The results of immunofluorescence staining showed that *Mvh* was expressed in the cells that treatment with 12.5 ng/ml BMP4. The cells characterized as GCLCs exhibited round nuclei, as shown by Hoechst nuclei counter stain in the upper slide. Also, immunohistochemistry examination showed that *Mvh* was expressed in tissue section of testis as positive control lower slide (Figure 5).

**Flowcytometry**

**Figure 6:**
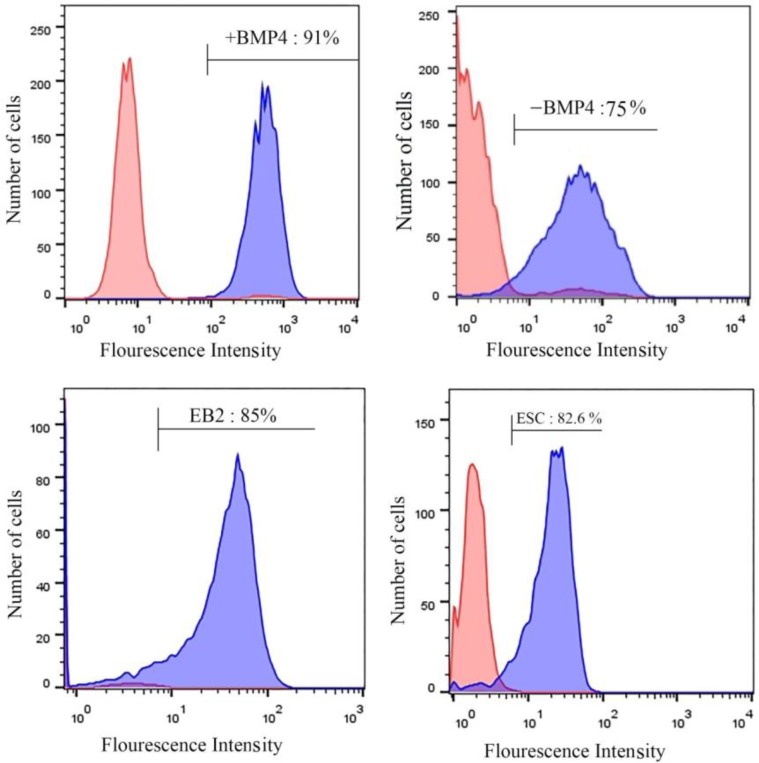
Flowcytometric analyses in study groups. Flowcytometric analyses show the expression of *Mvh* protein in the ESC (82.6±1.6), +BMP4 (91.3±3.5), -BMP4 (75±1.4) and EB2 (85±5.2) cells. *Mvh* expression in +BMP4 was more than other groups. Undifferentiated ESC as (ESC), ESC-derived GCLCs at the day 4 of culture with 12.5 ng/ml (+BMP4); Culture of day 2 of EB without BMP4 for 4 days control group (-BMP4); ESCs differentiation after 2 days in suspension culture as embryoid body (EB2).

Since the real-time RT-PCR showed that the expression of germ cell markers enhanced in differentiated cells, we investigated the protein expression of *Mvh* by flowcytometry analysis. The mean fluorescent intensity (MFI) of the cells for *Mvh* showed that more *Mvh* positive cells were observed in +BMP4 group (91.3±3.5), compared to that observed in the undifferentiated ESCs (82.6±1.6), (p=0.03). In –BMP4 group, MFI was (75±1.4) compared with +BMP4 group (p=0.001). In EB2, MFI was (85±5.2) compared with –BMP4 (p=0.02) (Figure 6).

## Discussion

This study demonstrated that several GC-specific genes (*Fkbp6*, *Tex13*, *Mov10l1*, *4930432K21Rik*) exhibited differential expression profiles in ESCs, GCLCs, and somatic tissues. These genes have low expression in ESCs, and their levels increase in both male and female germ cells (20). We showed that BMP4 12.5 ng can increase the expression of the germ cells genes compared to the control group, which in some cases was significantly more than the control group. It was shown that in bone mesenchymal stem cells-derived PGCs, 25 ng/ml BMP4 had a better effect on the expression of the VASA compared with another dose (21). Also, Aflatoonian and colleagues showed that in hESCs BMP4 with a dose of 10 ng/ml at day 14 had a greater effect on the expression of the VASA compared to day four and seven of culture. They also suggested that RA compare to BMP4 10 ng/ml was a more effective inducer of hESCs to form post-meiotic spermatids in vitro (22). BMP4 is known to control cell growth and development. It is a mitogen factor in in vitro cultured PGCs (23). 

There is also evidence that suggests in EB-derived human after three weeks cell culture BMP4 has increased VASA expression (12). The EB between days 2-3 consists of an outer layer of primary endodermal cells that surrounds the ectodermal cells, which forms a solid core that is equivalent to cells of the epiblast in the embryo. In vivo, the ectodermal and endodermal cells produce BMP4 (24) and BMP2, respectively; this mediates differentiation of the primary endoderm into visceral endoderm after 3-4 days. It plays a role in the induction of PGC precursors (25). Based on this model differentiation of the structure like this, in EBs cultured with BMP4 can stimulate expression of GC markers in vitro. *Mvh* is a special marker for germ cells. This gene is expressed in post-migratory PGCs, but not expressed in ESCs (26). The various effects of short-and long-term BMP4 treatment on human stem cells suggest that the BMP4 signaling pathway may play a flexible and time-dependent role in human embryo development and fate determination (14). 

In this study, the expression of the *Mov10l1* gene in BMP4-treated cells was increased, but the difference was not significant as compared with the control. *Mov10l1* is an X-linked and GC-specific gene that is expressed in mouse spermatogonial cell (17). Its expression was increased in the pachytene stage during first meiosis, where spermatogonia-a differentiated into spermatocytes. In vivo studies demonstrated that *Mov10l1* expression in male mouse fetal PGCs of embryonic day (E) 12.5, in contrast to that of females, begins to increase and gradually decreases until E18.5. It has been shown to exhibit the greatest expression in adult mouse testis and the least expression in sperm cells (20). 

The present study showed that *Tex13* was expressed in the +BMP4 groups. This gene is usually expressed in adult testis, matured spermatozoa and male mouse fetal PGCs at E12.5 (18). *Tex13* is an X-linked gene expressed in early spermatocytes, during the liptoten and zigoten stages of first meiosis. However, it seems to undergo translational suppression before late meiosis (27). Our results showed that expression of *Fkbp6* was increased in the +BMP4 and -BMP4 groups, as well as in the EB2 group but there are no significant differences between them. Mouse *Fkbp6* is not involved in the initiation of synapsis but does play a role in monitoring progression and/or maintaining synapsis between homologous pairs. Accordingly, *Fkbp6* is a component of the synaptonemal complex, and is essential for sex-specific fertility and further facilitates the pairing of homologous chromosomes during meiosis (28). However, its expression was demonstrated to be drastically reduced in sperm, suggesting that it may a major role in the creation of a synaptic complex in the paired homologous chromosome (20). 

In this study, Riken expression was increased in the +BMP4 group, with significant differences compared to the -BMP4 group. Riken is a new gene with unknown function. This gene is expressed in PGCs of mouse embryos, adult testis, spermatozoa, and oocytes. There are not many reports on this gene (20). Further to studying four GC-specific genes, we were also studied four other genes that generally are expressed in GCs. In this study, expression levels of *Mvh* were greater in the +BMP4 group as compared with those in the -BMP4 and EB2 groups. The expression of this gene is limited to the germ cell line. Spermatozoa terminate in the first meiosis without *Mvh* (29). The expression of this gene increases with the onset of the meiosis and remains high until the end of spermatogenesis (30). *Mvh* expression at the protein level, as determined by immunofluorescence staining and flowcytometry, was higher in the +BMP4 group as compared with that in the control group, which confirmed the findings of real-time RT-PCR. Immunohistochemical studies have been shown that the *Mvh* protein in PGCs was expressed exclusively following embryonic gonadal colonization on E12.5 (31). 

In this study, *Oct4* gene expression decreased in all studied groups compared with that in ESCs. During gastrulation and stem cell differentiation, *Oct4* expression decreases and is limited to ESCs. In vivo studies showed that GCs exhibit a high expression of *Oct4* until E13.5 then decreases in the zygote/pachytene of first meiosis around E16.5 (32). In this study, the expression level of *Scp3* (synaptonemal complex protein 3) in the +BMP4 group was increased with significant differences as compared with those in the EB2, but not significant with–BMP4 group. *Scp3* is essential for the synaptonemal complex formation, chromosomal synapse, and male fertility. Male mice that do not have *Scp3*, exhibit defective formation of axial/lateral elements and chromosomes that lack this gene cannot form a synaptic complex (33). 

In our study, *Stra8* expressed in differentiated cells. This increase was significant between +BMP4 and EB2 but not significant with –BMP4. *Stra8* expressed in a higher level than adult mouse testis (positive control). Expression of this gene is limited to non-differentiated spermatogonia. It has been shown that expression of the *Stra8* gene is initially present in immature testis and in GCs with mitotic activity after birth; then increased in undifferentiated GCs of the adult testis. It is essential for initiation of meiosis (34). 

## Conclusion

According to our findings, *Tex13*, Riken, *Fkbp6*, *Mvh* as germ cell, *Scp3* and *Stra8 *as meiosis markers were expressed in +BMP4 group while *Mvh* and Riken expression levels were increased with significance differences compared with control group. On the other hand, our result showed that expression levels of *Oct4* as a pluripotency factor decreased in all groups. Immunofluorescence staining showed the protein expression of *Mvh* in differentiated cells as well as flowcytomerty analysis. So, induction of mESCs by 12.5ng/ml BMP4 may cause differentiation towards GCLCs. Improving in vitro germ cell differentiation with high efficiency may simplify the generation of mature gametes for an understanding of the biology of gametogenesis. 
